# MRI based neuroanatomical segmentation in breast cancer patients: leptomeningeal carcinomatosis vs. oligometastatic brain disease vs. multimetastastic brain disease

**DOI:** 10.1186/s13014-019-1380-3

**Published:** 2019-09-18

**Authors:** Michael Mayinger, Antonia Reibelt, Kai Joachim Borm, Johannes Ettl, Jan J. Wilkens, Stephanie Elisabeth Combs, Markus Oechsner, Marciana Nona Duma

**Affiliations:** 1 Department of Radiation Oncology, Medical School, Klinikum rechts der Isar, Technical University of Munich, Munich, Germany; 2 Department of Radiation Oncology, University of Zurich, Zurich, Switzerland; 3 Department of Obstetrics and Gynecology, Klinikum rechts der Isar, Technical University of Munich, Munich, Germany; 4 Deutsches Konsortium für Translationale Krebsforschung (DKTK)-Partner Site Munich, Munich, Germany; 5 Institute of Innovative Radiotherapy, Helmholtzzentrum München, Munich, Germany; 6 Department of Radiotherapy and Radiation Oncology, University Hospital of the Friedrich-Schiller-University, Bachstr. 18, 07745 Jena, Germany

## Abstract

**Purpose:**

Pathogenesis of brain metastases/meningeal cancer and the emotional and neurological outcomes are not yet well understood. The hypothesis of our study is that patients with leptomeningeal cancer show volumetric differences in brain substructures compared to patients with cerebral metastases.

**Methods:**

Three groups consisting of female breast cancer patients prior to brain radiotherapy were compared. Leptomeningeal cancer patients (LMC Group), oligometastatic patients (1–3 brain metastases) prior to radiosurgery (OMRS Group) and patients prior to whole brain radiation (WB Group) were included. All patients had MRI imaging before treatment. T1 MRI sequences were segmented using automatic segmentation. For each patient, 14 bilateral and 11 central/median subcortical structures were tested. Overall 1127 structures were analyzed and compared between groups using age matched two-sided t-tests.

**Results:**

The average age of patients in the OMRS group was 60.8 years (± 14.7), 65.3 (± 10.3) in the LMC group and 62.6 (± 10.2) in the WB group. LMC patients showed a significantly larger fourth ventricle compared to OMRS (*p* = 0.001) and WB (*p* = 0.003). The central corpus callosum appeared smaller in the LMC group (LMC vs OMRS *p* = 0.01; LMC vs WB *p* = 0.026). The right amygdala in the WB group appeared larger compared with the OMRS (*p* = 0.035).

**Conclusions:**

Differences in the size of brain substructures of the three groups were found. The results appear promising and should be taken into account for further prospective studies also involving healthy controls. The volumetrically determined size of the fourth ventricle might be a helpful diagnostic marker in the future.

## Introduction

Brain metastases (BM) are the most common intracranial tumors in adults affecting 20 to 40% of all cancer patients [1–3]. BM occur in about 5% of patients with breast cancer (BC) at some point in the course of their disease [4–6]. The one-year survival rate of patients with parenchymal BM is about 50% [7]. Prospective trials have helped to guide treatment decisions for BM [8] and retrospective reviews have identified factors such as number of metastases, the presence or absence of active systemic disease, and hormone receptor status as having an impact on survival [9, 10].

Leptomeningeal cancer (LMC) is a rare, but often devastating form of tumor spread [5, 11]. The most common solid tumors leading to LMC are BC and lung cancer [12–14]. In particular women with invasive lobular carcinoma of the breast have a predilection to metastasize to the leptomeninges [15–17]. LMC patients can present a broad range of symptoms due to the involvement of multiple areas of the craniospinal axis. Diagnosis often requires a high index of suspicion and is confirmed by neuroimaging and cerebrospinal fluid analysis. Coexisting BM are present in 50 to 80% of LMC patients [14, 18–21]. Despite improvements in the treatment of BC, outcomes of BM and especially LMC remain unsatisfactory. An analysis of 36 studies including 851 patients reports a median survival of BC patients with LMC of 15 weeks [22], the 1-year survival varies from 7 to 24% [23–31].

Despite the devastating prognosis, the intrinsic brain changes and pathogenesis of LMC/BM are not yet well understood. The aim of our study was to compare volumetric differences in brain substructures of breast cancer patients with oligo−/multiple parenchymal brain metastases/LMC.

## Material and methods

We selected breast cancer patients treated between 2011 and 2017 for leptomeningeal cancer and parenchymal brain metastasis from our database. All institutional guidelines were followed. Informed consent was obtained from all patients. Bavarian state law (Bayrisches Krankenhausgesetz §27 Abs. 4 Datenschutz) allows the retrospective use of patient imaging data for research and publication, provided that any personal related data are kept anonymous. No patient received cranial radiotherapy or surgery prior to the treatment. Thirty age matched patients (10 in each group) were included in this retrospective analysis. To minimize heterogeneity within groups, due to gender differences in brain volume, only female patients were included in this study.

The three groups were:

- leptomeningeal cancer patients (LMC group)
- oligometastasic patients (1–3 brain metastases) (oligometastatic radiosurgery group – OMRS group) and
- multiple metastatic brain patients (whole brain group – WB group).

Table 1 depicts the patients’ characteristics including age, luminal stage and Karnofsky Index.

**Table 1:** Patients’ characteristics

	LMC	OMRS	WB
Age	65,3 (± 10.3)	60,8 (± 14.7)	62,6 (± 10.2)
Luminal A	0	0	0
Luminal B	7	1	2
Luminal B/Her 2 +	0	6	4
Her 2 +	0	0	0
Triple negative	1	2	4
unknown	2	1	0
Time since diagnosis (months)^a^	88 (15–163)	82.5 (18–182)	21 (2–194)
Extracranial metastasis (x patients out of 10 patients: x/10)	8/10	6/10	7/10
Karnofsky Index^a^	70% (40–80%)	80% (60–100%)	70% (40–80%)
Initial side of primary cancer:			
left sided	7	5	7
right sided	3	4	3
Both-sided		1	

^a^median (minimum-maximum)

**Fig. 1:**
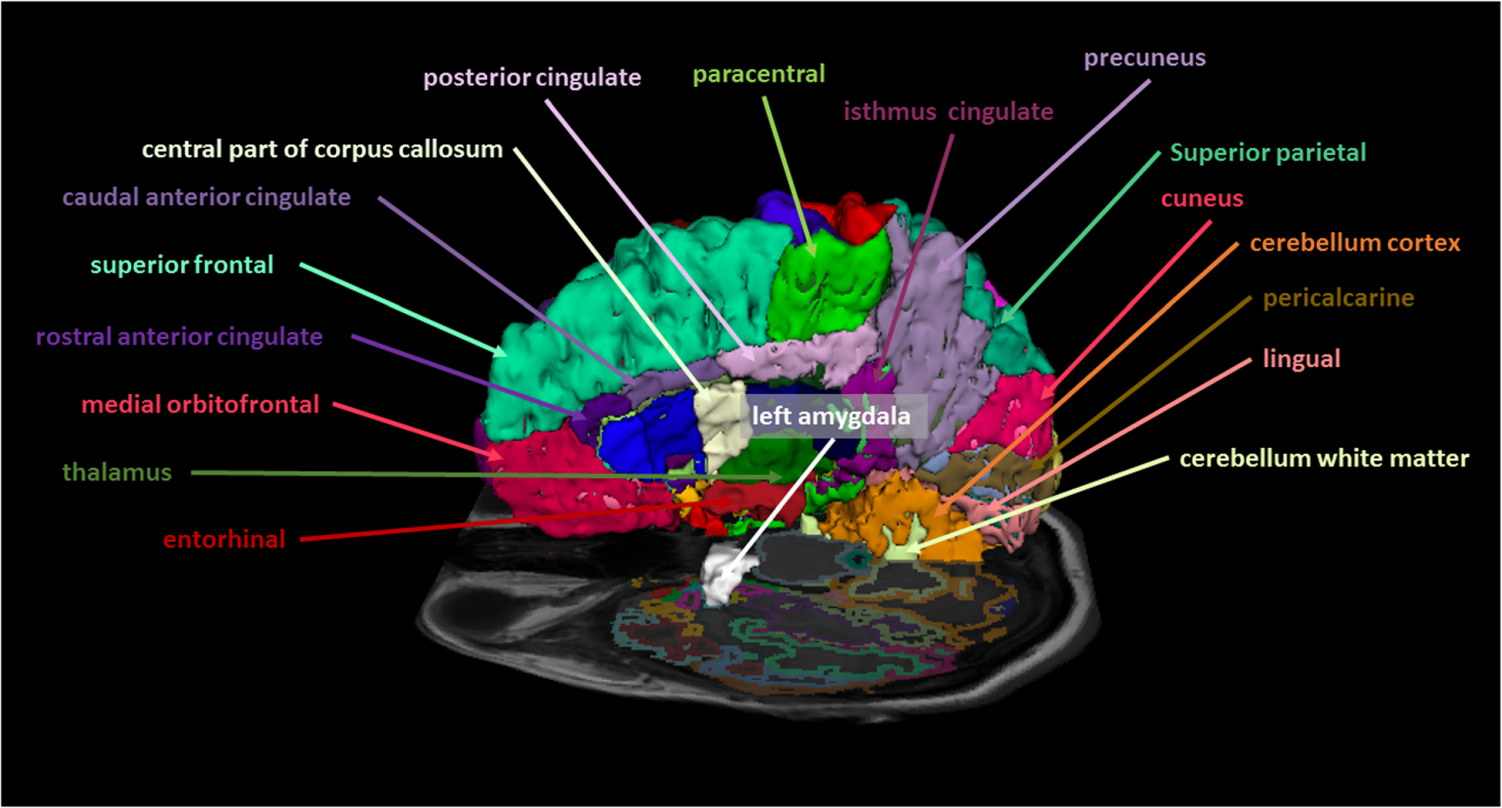
Exemplary depiction of some of the segmented structures

This retrospective study was approved by the Local Ethics Committee. All patients received magnetic resonance imaging (MRI) in a supine position before treatment**.** Data acquisition was performed on a 3 T MR scanner (Magnetom Verio, Siemens Healthcare, Erlangen, Germany) with a 32-channel head coil array. The scanning protocol included a T1-weighted 3D magnetization rapid-acquisition gradient echo (MP-RAGE) acquired in an axial orientation. The T1- weighted images were transformed from the DICOM to NRRD file format by creating an nhdr header file for each subject. Cortical thickness analysis was performed using FreeSurfer version 5.3 (Athinoula A. Martinos Center for Biomedical Imaging, Charlestown, MA, USA). Cortical reconstruction and volumetric segmentation were performed with the FreeSurfer image analysis suite (http://surfer.nmr.mgh.harvard.edu/). The technical details of these procedures are described elsewhere [32, 33]. The images were aligned to a common atlas and the grayscale intensity was normalized and corrected for inhomogeneity of the magnetic field. All voxels were labeled as gray matter, white matter or cerebral spinal fluid and the gray matter surface (pial) and white matter surface were created. The deep grey matter in each hemisphere was segmented into seven subcortical structures. Cortical surface was parcellated into discrete units based on gyral and sulcal anatomy (Fig. 1). In case of segmentation inaccuracies, e.g. caused by edema or lesions close to structural borders such as grey/white matter, manual correction was performed. Two out of thirty scans required manual correction. In all other cases FreeSurfer performed well and labelled even small BMs as “area of unknown origin”.

Using this approach none of the structures had to be excluded. A detailed list of all structures analyzed is provided in the Table 2. Statistical analyses were performed in SPSS 22 (SPSS, Chicago, IL). Overall 1127 structures were analyzed and compared within the three groups using age matched two-sided t-tests: When performing the two - sided t-test between groups (e.g.: LMC compared to OMRS) every patient in one group (e.g.: LMC) was matched by age to one person of the other group (e.g.: OMRS). Corresponding surface analyses were performed for each hemisphere. Results were adjusted with the Bonferroni correction for multiple region comparisons (*P* < 0.001 after correction was considered statistically significant).

**Table 2:** Volumes of segmented cerebral structures (in mm^3^)

Cerebral structure	LMC	OMRS	WB
Left - Lateral - Ventricle	11,595.5 (±5050.4)	11,422.6 (±4343.2)	10,472.7 (±6418.9)
Left - Inf - Lateral - Ventricle	325.4 (±302)	248.7 (±354.8)	236.2 (±228.9)
Left - Cerebellum - WM	16,322 (±4892.6)	16,101 (±4047)	13,873.3 (±4701.1)
Left - Cerebellum - Cortex	49,269.7 (±5394.7)	44,018.6 (±8607.7)	40,789.9 (±12,591.2)
Left - Caudate	3077.1 (±1042.8)	2524.8 (±485.6)	2589.1 (±766.3)
Left - Putamen	4375.8 (±1590.6)	4325.7 (±1059.1)	4937.7 (±707.8)
Left - Pallidum	1883.6 (±314.9)	1963.9 (±510.6)	2081.9 (±381.1)
3rd - Ventricle	1594.4 (±633.8)	1400.4 (±485.3)	1368.7 (±697.7)
Brain - Stem	20,123.6 (±1994.7)	20,181 (±3517.8)	19,878.4 (±5098)
Left - Hippocampus	3652.6 (±779.1)	3519.3 (±723.9)	3778.4 (±808.6)
Left - Amygdala	1294 (±273.6)	1184.1 (±231.7)	1163.5 (±357)
CSF	1155.9 (±362.8)	1540.6 (±545)	1388.8 (±575.5)
Left - Accumbens - area	323.2 (±96.5)	306 (±102.2)	402.1 (±133)
Left - Ventral DC	3961.7 (±525.6)	4469.9 (±770.5)	4738.7 (±930.7)
Left - vessel	12.9 (±27.5)	12,970.5 (±20,869.8)	8664.9 (±18,248.5)
Left - choroid - plexus	594.8 (±199.2)	575.2 (±236.4)	411.9 (±276.1)
Right - Lateral - Ventricle	11,245.6 (±4532)	9628.1 (±4296.2)	8272.1 (±4300)
Right – Inf – Lateral - Ventricle	532.6 (±458.2)	238.8 (±369.8)	277.3 (±207.6)
Right - Cerebellum - WM	13,878.7 (±3981.6)	15,196.4 (±3860.6)	12,392.2 (±4542.2)
Right - Cerebellum - Cortex	50,352.9 (±6280.5)	44,821.1 (±10,025.9)	42,623.8 (±12,097.7)
Right - Thalamus	6828.4 (±1277.9)	7788 (±1802.7)	8335.7 (±1550.8)
Right - Caudate	2930.6 (±728.9)	2662.7 (±462.8)	2568.9 (±763.1)
Right - Pallidum	1712.6 (±364.4)	1946.1 (±497.2)	1995.1 (±260.2)
Right - Accumbens - area	373.5 (±84.4)	364.4 (±104.8)	389.1 (±126.1)
Right - Ventral DC	3848.3 (±433.1)	4499.7 (±887.3)	4533.9 (±791.2)
Right - vessel	18.7 (±42.9)	8662.7 (±18,240)	25,980.3 (±22,352.6)
WM - hypointensities	49,110.5 (±42,658)	52,212 (±30,669.8)	57,353.5 (±36,403.1)
Optic - Chiasm	152.5 (±76.4)	154.7 (±94.3)	140.9 (±98.7)
CC_Posterior	1179.8 (±292.3)	1484.5 (±508.3)	1546.5 (±474.2)
CC_Mid_Anterior	919.4 (±609.5)	1034.8 (±458.8)	1262 (±375.1)
CC_Anterior	2454.4 (±4659.7)	1261.4 (±372.1)	1162.7 (±225.1)

*CC* Corpus callosum, *CSF* Cerebrospinal fluid, *DC* Diencephalon, *Inf* Inferior, *WM* White matter

## Results

The average age in the OMRS group was 60.8 years (± 14.7), 65.3 (± 10.3) in the LMC group and 62.6 (± 10.2) in the WB group.

All analyzed structures are available in the Table 2. It shall be noted that after Bonferroni correction the results for the fourth ventricle remained statistical significant. Other findings have to be interpreted with caution, as these may have occurred due to chance.

In the **LMC** group several analyzed subcortical structures showed an increased size in comparison to either the OMRS and/or WB group. LMC patients showed a significantly larger fourth ventricle compared to both the OMRS and WB group. Further, the central corpus callosum was significantly smaller compared to both, the OMRS and the WB group. The right hippocampal volume and the right choroid plexus were also significantly increased in LMC as compared to the OMRS group.

Looking at the subcortical structures of the **OMRS** group the central corpus callosum was shown to be significantly larger in comparison with the LMC group.

The **WB** group showed an increased size in several subcortical substructures in comparison with the LMC and/or OMRS group: the central corpus callosum, the mid posterior part of the central corpus callosum, the left thalamus and the right putamen were significantly larger compared with the LMC group. Furthermore, the right amygdala showed an increased size compared with the OMRS group.

**Fig. 2:**
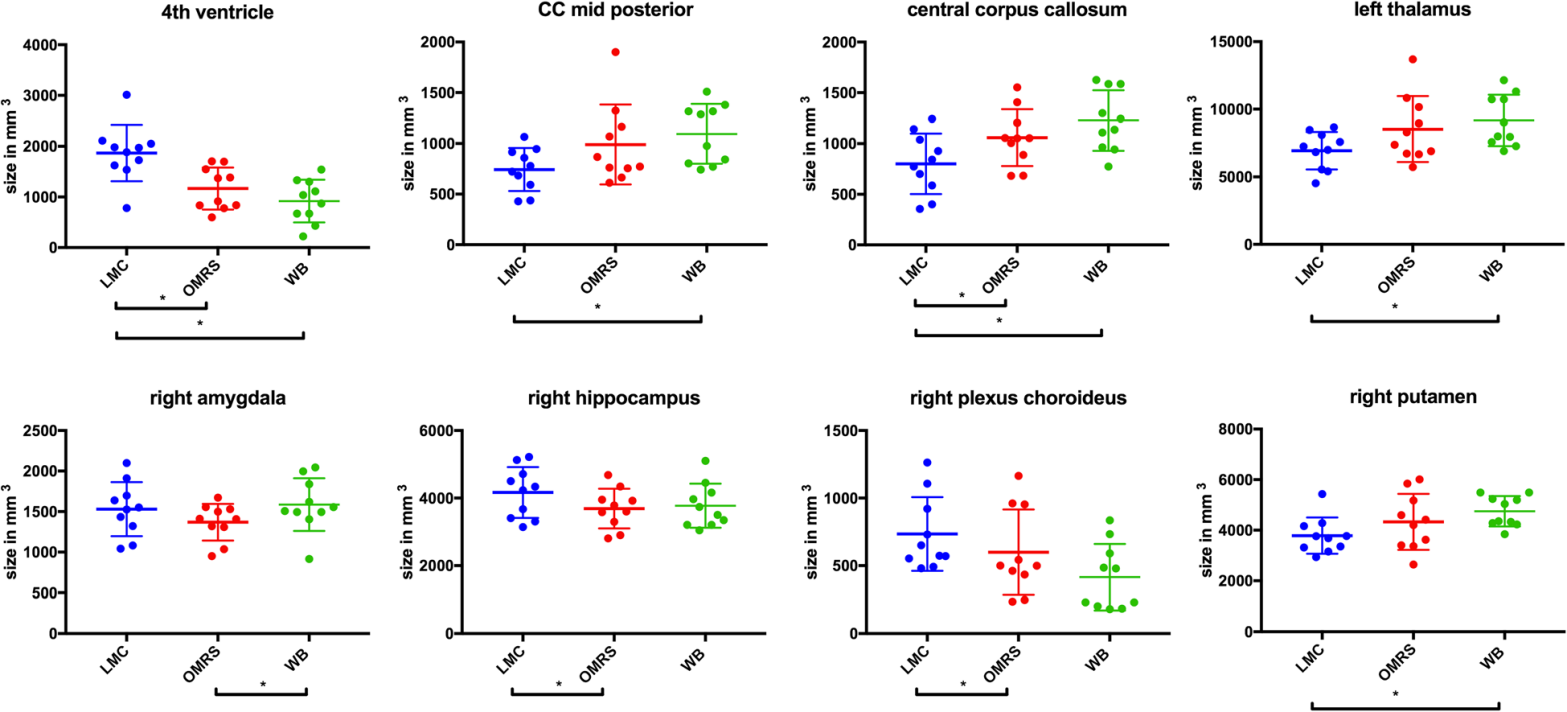
Structures with significant differences between groups

All significant differences amongst groups are depicted in Fig. 2.

## Discussion

Image segmentation is often the first step in clinical applications. It is commonly used for measuring and visualizing the brain’s anatomical structures, for analyzing brain changes, for delineating pathological regions and for surgical planning and image-guided interventions.

This is the first study to evaluate volumetric neuroanatomical differences in the gray and white matter of BC patients. We found several differences between groups of differing forms of metastatic spread.

The LMC group showed an increased size of the fourth ventricle compared to the WB and OMRS groups. According to DeAngelis et al. leptomeningeal cancer invades the base of the brain and occludes the cerebrospinal fluid outflow of the fourth ventricle. Further, the meningeal tumor and its accompanying inflammatory response also reduce the cerebrospinal fluid absorption. Thus, intracranial pressure is slowly elevated and may result in ventricular dilatation [34]*.* Jung et al. have shown that surgically treating hydrocephalus in patients with leptomeningeal cancer can improve the overall survival [35]. Brain segmentation, as demonstrated by our study, point out the ventricular dilatation and could be used in a clinical setting to identify early hydrocephalus in leptomeningeal cancer patients and potentially improve patients’ prognosis. Leptomeningeal cancer can be difficult to prove. Often several biopsies are needed, in order to detect tumor cells in the cytology. In this study, 4 out of ten patients in the LMC group received cerebral spinal fluid (CSF) puncture. Out of these 4 patients, 2 had tumor cells in their CSF. All other patients were diagnosed by cranial MRI imaging and neurological symptoms. An enlarged fourth ventricle, as shown by our study, may provide another diagnostic tool of leptomeningeal cancer, especially in cases with negative liquor cytology.

The central corpus callosum, which connects the left and right cerebral hemispheres, was found to be significantly smaller in the LMC group compared with the WB and OMRS groups. Research using mice with either meningeal overgrowth or selective loss of meninges, has identified a cascade of morphogenic signals initiated by the meninges that regulates corpus callosum development. BMP7, produced either by overexpression in the medial cortical wall or by hyperplastic meninges, is sufficient to cause callosal agenesis [36]. This suggests a link between leptomeningeal cancer leading to an overexpression of certain signals and the decreasing size of the central corpus callosum. In addition, more research is starting to link corpus callosum decline to cognitive decline. A study on cognitive impairment in MS patients states “Corpus callosum atrophy predicts a clinically meaningful cognitive decline, … “[37]. Similarly, other studies on bipolar disorder and suicidal vulnerability as well as major depression and dementia are pointing to corpus callosum volume decrease, playing a central role in neurocognitive dysfunction [38–40].. Hence, corpus callosum atrophy may be hold responsible for the quick cognitive decline in leptomeningeal cancer patients and should be addressed further by future research on clinical manifestations of leptomeningeal cancer. However, it needs to be noted that degeneration of corpus callosum in MS and dementia is a long lasting atrophic process. Rapid decrease of corpus callosum in LMC patients appears somewhat peculiar and could also be related to raised intracranial pressure.

The right amygdala, which has a direct correlation with negative emotions, especially fear and sadness, was shown to be larger in the WB group in comparison with the OMRS group [41]. Although serious depression is not seen in the majority of breast cancer patients and survivors, many experience treatment-related distress and fear of recurrence [42]. Earlier studies have shown that psychosocial stress can affect inflammatory processes that have important consequences for cancer outcomes [43, 44]. Muscatell et al. reported a strong, positive correlation between circulating inflammatory markers concentration in response to stress and amygdala activity [45, 46]. The increased size of the right amygdala can be interpreted as an increased stress reaction and /or inflammatory response reaction and could be limited with psychological interference [47, 48]. Although these findings might suggest higher anxiety levels in LMC patients it is very difficult to interpret these results in absence of a healthy control group.

Our results showed an increased volume of the right hippocampus in the LMC group in comparison with the OMRS group. Research investigating the hippocampus, which is involved in creating and organizing new memories, has shown that morphologic differences exist between the right and left hippocampus in animal testing [49]. Research on the individual role of the right and left hippocampus is not yet conclusive, but should be performed, especially when WBRT with hippocampal sparing is on its way as a new and promising treatment.

The right choroid plexus was significantly increased in the LMC group in comparison with the OMRS group. The choroid plexus is a plexus of cells that produces the cerebrospinal fluid in the ventricles of the brain [50]. So far, a distinction between the different parts of the choroid plexus in brains of patients with metastases has not been investigated.

The left thalamus and the right putamen in the WB group were also significantly larger compared to the LMC group. The thalamus has several functions such as relaying sensory signals and the regulation of consciousness, sleep, and alertness. Studies investigating language deficiencies have shown that the right and left Thalamus are not functionally equivalent [51]. A connection between differing thalamus sizes of the two hemispheres and the occurrence of meningeal cancer has not been examined.

The primary function of the putamen is to regulate movements and influence various types of learning. Using MRI of 98 individuals (male and female) of various ages no hemispherical asymmetry in putamen volume has been found [52].

As of today, our knowledge concerning hemispherical differences of subcortical structures such as the hippocampus, the choroid plexus, the thalamus and the putamen is not sufficient to discuss and formulate perfect explanation to our results. The right hippocampus and the right plexus choroid showed an increase in size in group LMC compared to OMRS. Also, the right amygdala showed an increase in group WBR compared to group OMRS. Yet these are only observations with no sufficient data to support a hypothesis.

It has to be noted, that our results have to be interpreted cautiously in absence of an age matched healthy control group that would be helpful to define values as abnormal and to draw conclusions on effects of LMC and BM on size of brain structures.It is important to understand that our findings concerning the right hippocampus, left thalamus and right putamen should be interpreted with caution and might be due to artefacts. Nonetheless, it would be extremely interesting to validate these results by comparing it to a healthy control. Our sample size was small and the data was cross-sectional. As such it is unclear if the relation between volumetric neuroanatomical differences was present prior to cancer diagnosis or resulted from the cranial cancer manifestation. Future studies will need larger samples and an experimental longitudinal design.

## Conclusions

Several differences in size of the brain substructures were found. Most significantly fourth ventricle was enlarged in LMC, which might aid the diagnosis of LMC in the futureSize differences of corpus callosum and choroid plexus might also be related to direct or indirect effects of LMC.Differences in size of amygdala, thalamus, putamen and hippocampus need to be further validated in comparison to healthy control groups to better evaluate possible disease related effects on separate brain structures.

## Data Availability

The datasets used and/or analysed during the current study are available from the corresponding author on reasonable request. Part of the data was presented in October at the annual meeting of the American society of Radiation Oncology (ASTRO 2018) - accepted abstract no. MO_11_2559; Track: Central Nervous System.

## Abbreviations

BM: Brain metastases
CN: Caudate nucleus
GP: Globus pallidus
LM: Leptomeningeal metastasis
LMC Group: Leptomeningeal cancer patients
OMRS Group: Oligometastasic patients prior to radiosurgery
PT: Putamen
TH: Thalamus
WB Group: Whole brain group
